# Prevalencia de anticuerpos contra el SARS-CoV-2 según el estatus socioeconómico y étnico en una encuesta nacional de Brasil^*^

**DOI:** 10.26633/RPSP.2021.105

**Published:** 2021-10-18

**Authors:** Bernardo L Horta, Mariângela F Silveira, Aluísio J D Barros, Fernando C Barros, Fernando P Hartwig, Mariane S Dias, Ana M B Menezes, Pedro C Hallal, Cesar G Victora

**Affiliations:** 1 Universidad Federal de Pelotas Pelotas Brasil Universidad Federal de Pelotas, Pelotas, Brasil.

**Keywords:** Epidemiología, infecciones por coronavirus, encuestas y cuestionarios, inequidad social, Brasil, Epidemiology, coronavirus infections, surveys and questionnaires, social inequity, Brazil, Epidemiologia, infecções por coronavirus, inquéritos e questionários, iniquidade social, Brasil

## Abstract

**Objetivos.:**

Investigar las desigualdades socioeconómicas y entre distintos grupos étnicos en la prevalencia de anticuerpos contra el SARS-CoV-2 en las 27 unidades federativas de Brasil.

**Métodos.:**

En este estudio transversal, se realizaron tres encuestas de hogares los días 14-21 de mayo, 4-7 de junio y 21-24 de junio de 2020 en 133 áreas urbanas brasileñas. Se utilizó un muestreo de etapas múltiples para seleccionar 250 individuos en cada ciudad a fin de someterlos a una prueba rápida de anticuerpos. Los sujetos respondieron un cuestionario sobre los bienes del hogar, la escolaridad y el color de la piel y etnia (autodeclarado utilizando la clasificación brasileña estándar de cinco categorías: blanco, negro, pardo, asiático o indígena). Se utilizó el análisis de los componentes principales de los bienes para clasificar el estatus socioeconómico en cinco quintiles de riqueza. Se empleó la regresión de Poisson para los análisis.

**Resultados.:**

Se analizaron 25 025 sujetos en la primera encuesta, 31 165 en la segunda y 33 207 en la tercera, que mostraron una prevalencia de resultados positivos de 1,4%, 2,4% y 2,9%; respectivamente. Los individuos del quintil más pobre tuvieron 2,16 veces más probabilidades de presentar un resultado positivo (intervalo de confianza del 95%: 1,86-2,51) que los del quintil más rico, y los que tenían 12 o más años de escolaridad tuvieron una prevalencia menor que los sujetos con menos educación. Las personas indígenas presentaron una prevalencia 4,71 (IC95%: 3,65-6,08) veces mayor que las blancas, al igual que las de piel negra o parda. El ajuste por región del país redujo los índices de prevalencia según la riqueza, la educación y el origen étnico, pero los resultados siguieron siendo estadísticamente significativos.

**Conclusiones.:**

La prevalencia de anticuerpos contra el SARS-CoV-2 en Brasil muestra gradientes relacionados con la posición socioeconómica y la etnia muy pronunciados, con menor riesgo en las personas blancas, educadas y ricas.

## Abstract (en)

**Objectives.:**

To investigate socioeconomic and ethnic group inequalities in prevalence of antibodies against SARS-CoV-2 in the 27 federative units of Brazil.

**Methods.:**

In this cross-sectional study, three household surveys were carried out on May 14-21, June 4-7, and June 21-24, 2020 in 133 Brazilian urban areas. Multi-stage sampling was used to select 250 individuals in each city to undergo a rapid antibody test. Subjects answered a questionnaire on household assets, schooling and self-reported skin color/ethnicity using the standard Brazilian classification in five categories: white, black, brown, Asian or indigenous. Principal component analyses of assets was used to classify socioeconomic position into five wealth quintiles. Poisson regression was used for the analyses.

**Results.:**

25 025 subjects were tested in the first, 31 165 in the second, and 33 207 in the third wave of the survey, with prevalence of positive results equal to 1.4%, 2.4%, and 2.9% respectively. Individuals in the poorest quintile were 2.16 times (95% confidence interval 1.86; 2.51) more likely to test positive than those in the wealthiest quintile, and those with 12 or more years of schooling had lower prevalence than subjects with less education. Indigenous individuals had 4.71 (3.65; 6.08) times higher prevalence than whites, as did those with black or brown skin color. Adjustment for region of the country reduced the prevalence ratios according to wealth, education and ethnicity, but results remained statistically significant.

**Conclusions.:**

The prevalence of antibodies against SARS-CoV-2 in Brazil shows steep class and ethnic gradients, with lowest risks among white, educated and wealthy individuals.

## RESUMO

**Objetivos.:**

Investigar as desigualdades socioeconômicas e étnicas na prevalência de anticorpos contra SARS-CoV-2 nas 27 unidades federativas do Brasil.

**Métodos.:**

Neste estudo transversal, três pesquisas domiciliares foram realizadas de 14 a 21 de maio, 4 a 7 de junho, e 21-24 de junho, 2020 em 133 áreas urbanas brasileiras. Amostragem em várias etapas foi utilizada para selecionar 250 indivíduos em cada cidade para se submeter a um teste rápido de anticorpos. Os sujeitos responderam a um questionário sobre bens domésticos, escolaridade e cor da pele/etnicidade (auto-relatada utilizando a classificação padrão brasileira de cinco categorias: branco, preto, pardo, asiático ou indígena). A análise dos componentes principais dos ativos foi utilizada para classificar a posição socioeconómica em cinco quintis de riqueza. A regressão de Poisson foi utilizada para as análises.

**Resultados.:**

25 025 indivíduos foram testados na primeira pesquisa, 31 165 na segunda, e 33 207 na terceira, com prevalência de resultados positivos de 1,4%, 2,4% e 2,9%, respectivamente. Indivíduos no quintil mais pobre tinham 2,16 vezes (intervalo de confiança de 95% 1,86; 2,51) mais probabilidade de ter um resultado positivo do que aqueles do quintil mais rico, e aqueles com 12 ou mais anos de escolaridade tinham uma prevalência menor do que aqueles com menos educação. Os indivíduos indígenas tinham 4,71 (3,65; 6,08) vezes mais prevalência do que os brancos, assim como aqueles com cor da pele preta ou parda. O ajuste regional reduziu as taxas de prevalência de acordo com a riqueza, educação e etnia, mas os resultados permaneceram estatisticamente significativos.

**Conclusões.:**

A prevalência de anticorpos contra a SARS-CoV-2 no Brasil mostra gradientes relacionados com a posição socioeconómica e a etnia muito acentuados, com os menores riscos entre os indivíduos brancos, educados e ricos.

La pandemia de COVID-19 está azotando a los países latinoamericanos con gran intensidad. Al 16 de septiembre del 2020, Brasil era el segundo país del mundo, después de Estados Unidos, con el mayor número absoluto de muertes (consultar mapa en: https://coronavirus.jhu.edu/map.html). Casi todos los días sobrevienen más de 1 000 muertes (https://covid.saude.gov.br).

Los casos destacados de COVID-19 en Brasil, como los de los gobernadores estatales y, más recientemente, el del Presidente Jair Bolsonaro (https://www.bbc.com/news/world-latin-america-53319517), difundieron la impresión de que la epidemia afecta a la sociedad brasileña en su conjunto, sin distinción de clases ni grupos étnicos. De ser cierto, habría un marcado contraste entre este hallazgo y los datos de los países de ingresos altos, donde la pandemia está afectando de manera desproporcionada a las minorías étnicas y a las poblaciones pobres (1). En Estados Unidos (https://www.nytimes.com/interactive/2020/07/05/us/coronavirus-latinos-african-americans-cdc-data.html), las personas afroestadounidenses y latinas se están viendo más afectadas por la incidencia de la enfermedad y la mortalidad que las personas blancas, según los casos notificados. En un estudio llevado a cabo en la red del Centro de Investigación y Vigilancia del Oxford Royal College of General Practitioners, se observó que es más probable que las personas de piel negra y las que residen en las zonas más desfavorecidas reciban un diagnóstico de infección por el SARS-CoV-2 (2). En otro estudio realizado en el Reino Unido (3), se reveló que la pertenencia a un grupo étnico distinto del blanco y las puntuaciones más altas de carencia estaban fuertemente relacionadas con una mayor mortalidad por COVID-19. En cambio, las grandes encuestas nacionales realizadas en España no encontraron que la nacionalidad o la educación fuesen factores de riesgo para la presencia de anticuerpos contra el SARS-CoV-2 (4).

Solo encontramos un estudio sobre las desigualdades étnicas o sociales con respecto a la COVID-19 en los países de ingresos bajos o medianos. Baqui et al. explican que, en Brasil, la mortalidad hospitalaria por COVID-19 es mayor en las personas con ascendencia negra o mixta que en las blancas (5). En un comentario sobre su artículo se argumenta, sin proporcionar nuevos datos, que las condiciones de vida de los brasileños pobres los harían más vulnerables a la morbilidad y mortalidad por COVID-19 (6); su estudio no incluyó un número suficiente de personas indígenas para los análisis.

No nos fue posible localizar estudios poblacionales de los países de ingresos bajos o medianos sobre las desigualdades sociales y étnicas con respecto a la morbilidad o la mortalidad debidas a la COVID-19. El presente análisis tiene por objeto determinar las desigualdades socioeconómicas y étnicas con respecto a la prevalencia de anticuerpos contra el SARS-CoV-2 en 133 ciudades centinela de todo Brasil, como parte del estudio EPICOVID-19 (www.epicovid19brasil.org).

## MÉTODOS

En el presente estudio transversal se realizaron tres encuestas serológicas poblacionales repetidas en 133 ciudades centinela ubicadas en las 27 unidades federativas de Brasil. Las ciudades incluyeron a Brasilia, las 26 capitales estatales y las ciudades más grandes de cada una de las regiones intermedias del país, según lo establecido por el Instituto Brasileño de Geografía y Estadística (IBGE). En cada ciudad se seleccionaron 25 sectores censales urbanos con probabilidad proporcional al tamaño y en cada sector se realizó un muestreo aleatorio en 10 hogares. Se incluyeron a todos los integrantes de cada hogar que participó de la muestra, y se seleccionó al azar a uno de ellos para realizarle la prueba. En caso de que la persona seleccionada se negara a proporcionar una muestra de sangre, se procedía a seleccionar al azar a otro miembro de ese mismo hogar. Si esta persona también se negaba, los entrevistadores se trasladaban al hogar ubicado a la derecha del seleccionado en un inicio, que también se elegía en caso de que los residentes del primer hogar no estuvieran en casa. En el presente artículo, se agruparon los datos de las tres rondas de encuestas realizadas del 14 al 21 de mayo, del 4 al 7 de junio y del 21 al 24 de junio del 2020. En las 250 personas seleccionadas en cada ciudad, los márgenes de error (aproximadamente dos errores estándar) correspondientes a las cifras de prevalencia estimadas de 2%, 5% y 10% fueron 1,77, 2,70 y 3,79 puntos porcentuales, respectivamente; y a nivel nacional, para un tamaño total de la muestra de 33.250, los márgenes de error correspondientes fueron de 0,15, 0,24 y 0,33.

Se utilizó la prueba de anticuerpos contra el SARS-CoV-2 de Wondfo (Wondfo Biotech Co., Guangzhou, China) para determinar la presencia de anticuerpos contra el SARS-CoV-2 en muestras de sangre obtenidas por punción digital. Cuando se llevó a cabo la primera encuesta, esta era la única prueba disponible en el país en grandes cantidades y el Ministerio de Salud facilitó más de 100 000 pruebas para el estudio. Permite detectar las inmunoglobulinas de los isotipos IgG e IgM específicos contra los antígenos del SARS-CoV-2 mediante la técnica de inmunocromatografía. El reactivo de la prueba se compone de partículas de oro coloidal recubiertas de antígenos recombinantes del SARS-CoV-2. Tras la introducción de la muestra de sangre, el complejo formado por el anticuerpo reactivo, el antígeno y el oro coloidal es capturado por los anticuerpos IgM e IgG humanos presentes en la línea (T) de la ventana del kit, lo que da lugar a la aparición de una línea de color oscuro. Las pruebas válidas se reconocen por la línea de control positiva (C) que aparece en la misma ventana del kit. Si la línea de control no es visible, la prueba se considera no concluyente, lo cual es poco común.

La prueba rápida se sometió a estudios de validación independientes que utilizaron la RT-PCR como técnica de referencia. Según el fabricante, la prueba rápida tiene una sensibilidad de 86,4% y una especificidad de 99,6% (https://en.wondfo.com.cn/product/wondfo-sars-cov-2-antibody-test-lateral-flow-method-2/). Un estudio de validación realizado por el Instituto Nacional de Control de Calidad en Salud (INCQS, Fundación Oswaldo Cruz, Río de Janeiro, Brasil) reveló una sensibilidad de 100% y una especificidad de 98,7%. Whitman et al. evaluaron 10 pruebas inmunocromatográficas diferentes (7) e informaron que la prueba de Wondfo tenía una sensibilidad de 81,5% y una especificidad de 99,1%. Otro estudio de validación realizado por nuestro grupo de investigación observó una sensibilidad de 77,1% y una especificidad de 98,0% (8). Al agruparse los resultados de los cuatro estudios de validación, ponderados por tamaño de la muestra, se ha estimado que la sensibilidad es de 84,8% (IC95%: 81,4-87,8) y la especificidad de 99,0% (IC95%: 97,8-99,7) (8).

Los participantes respondieron cuestionarios cortos que incluyeron información sociodemográfica (sexo, edad, escolaridad, color de la piel, tamaño del hogar y bienes del hogar), síntomas relacionados con la COVID-19, uso de los servicios de salud, cumplimiento de las medidas de distanciamiento social y utilización de mascarillas. Dado que en Brasil hay una amplia población multiétnica, la clasificación oficial brasileña reconoce cinco grupos étnicos basándose en la pregunta “¿En qué grupo se clasificaría por su color de piel o raza?”. Las cinco opciones de respuesta son blanco, pardo, negro, asiático e indígena. Se instruyó a los entrevistadores para que marcaran la opción “asiática” cuando los encuestados dijeran ser de ascendencia asiática, y la opción “indígena” cuando se refirieran a cualquiera de las numerosas poblaciones indígenas. La categoría “pardo” corresponde a una ascendencia mixta, incluidas la europea, africana e indígena. Este sistema está respaldado por el movimiento afrodescendiente, que aboga por el desglose de todos los datos estadísticos nacionales a fin de aumentar su visibilidad (9). La posición socioeconómica se determinó con un índice de riqueza procedente de los análisis de los principales componentes de los bienes del hogar (10). El primer componente se dividió en quintiles. La escolaridad alcanzada se registró como el grado más alto completado satisfactoriamente.

Los trabajadores de campo utilizaron tabletas electrónicas para grabar las entrevistas completas, anotar todas las respuestas y fotografiar los resultados de las pruebas. El cuestionario se administró antes de revelar el resultado de la prueba a los participantes. Se repitieron las pruebas no concluyentes y en 35 personas se obtuvieron resultados no concluyentes en la segunda prueba, que se consideraron valores faltantes. Todas las pruebas con resultados positivos o no concluyentes fueron leídas por un segundo observador, así como el 20% de las pruebas negativas.

La prueba también se realizó a los entrevistadores y en todos los casos los resultados fueron negativos; además, se les proporcionó equipo de protección individual que era desechado después de la visita a cada hogar.

Se utilizó la versión 15 del programa Stata^®^ para realizar los análisis. Las proporciones de pruebas con resultado positivo según la región, el sexo, los quintiles de riqueza, el grado de escolaridad y el color de la piel se compararon con la prueba de ji-cuadrado. En el caso de las variables ordinales, tanto en los análisis bifactoriales como en los multifactoriales se estimó el valor de *p* para la tendencia lineal y la heterogeneidad; se presenta el que tiene el valorde *p* más bajo. También se estratificaron los análisis de la seroprevalencia de acuerdo con las variables socioeconómicas por región del país (norte, noreste, sureste, sur y centro oeste) utilizando la regresión de Poisson con varianza sólida para estimar las tasas de prevalencia. Todos los análisis fueron ajustados para el diseño de muestreo por conglomerados con el prefijo svy.

La autorización ética se obtuvo del Comité Nacional de Ética de Brasil (número de proceso CAAE 30721520.7.1001.5313), con el consentimiento informado por escrito de todos los participantes adultos; en el caso de los menores, los padres o cuidadores proporcionaron el consentimiento por escrito, y los niños o adolescentes también firmaron formularios de aceptación si estaban alfabetizados. El conjunto de datos se almacena de forma anónima. Los casos positivos fueron notificados a los sistemas municipales de vigilancia de la COVID-19.

## RESULTADOS

En las tres rondas de la encuesta de seroprevalencia se realizó la prueba a 89 397 personas, y se excluyeron de los análisis a 35 personas con resultados inconclusos en la primera prueba y en su repetición. Por consiguiente, en el presente estudio se evaluaron 89 362 personas. Las tasas de respuesta en las tres rondas fueron 54,4%, 52,6% y 55,6%, debido principalmente al hecho de que toda la familia estaba fuera de casa cuando tenían lugar las visitas. En la primera, segunda y tercera rondas la prevalencia de resultados positivos fue de 1,4%, 2,4% y 2,9%, respectivamente.

En el cuadro 1, se muestra que la proporción de hombres y jóvenes en la población estudiada estuvo por debajo de lo esperado sobre la base de la población nacional. En cuanto al color de la piel, la mayoría de las personas entrevistadas informaron pertenecer a la población parda o blanca y solo el 1,4% se autodeclaró indígena. La proporción de personas que informaron pertenecer a la población blanca fue menor que las estimaciones nacionales.

En las tres etapas del estudio hubo 2 064 pruebas con resultado positivo (2,31%) entre los 89 362 participantes con resultados válidos. En el cuadro 2, se observa que la proporción de pruebas con resultado positivo fue mayor en la región norte (6,7%), mientras que en la región Sur solo el 0,2% de los participantes dieron positivo en la prueba.

Los resultados de los análisis no ajustados y de los análisis ajustados por edad y sexo fueron muy similares. Se observó una relación inversa entre la prevalencia de anticuerpos y los quintiles de riqueza; la probabilidad de presentar anticuerpos contra el SARS-CoV-2 fue unas dos veces mayor entre las personas más pobres que entre las más ricas. En cuanto a la escolaridad, la relación no fue lineal, pero las personas con 12 o más años de estudio fueron menos propensas a dar positivo en la prueba que cualquiera de los otros grupos. El cociente de prevalencia más elevado (casi cinco veces más) se observó entre las personas indígenas y blancas. La probabilidad de dar positivo en la prueba fue menor en las personas blancas, seguidas de las asiáticas, por comparación con cualquier otro grupo étnico.

Dado que la proporción de personas con anticuerpos contra el SARS-CoV-2 fue mayor en la región Norte (Amazonas), donde se concentran las poblaciones indígenas y pobres, se realizaron análisis complementarios con otro ajuste para las cinco regiones del país. En estos análisis, la prevalencia de la seropositividad también fue menor en el quintil de los más ricos, pero la magnitud del cociente de prevalencia disminuyó. La prevalencia entre las personas indígenas siguió siendo mayor que entre las blancas (cociente de prevalencia 2,25; IC95%: 1,74-2,91), y este también fue el caso de las personas clasificadas en las categorías negro y pardo.

En el cuadro 3, se muestra que a pesar de la disminución del grado de asociación en la región Norte en comparación con los análisis nacionales (cuadro 2), la relación inversa con la riqueza siguió siendo significativa; asimismo, persistió la mayor prevalencia entre las personas indígenas y pardas en comparación con las blancas. En la región Noreste también se observó una relación inversa entre la seroprevalencia y la riqueza, pero no con el grupo étnico. Entre las personas indígenas la prevalencia fue 2,27 veces mayor que entre las blancas. En las demás regiones, donde la prevalencia era baja cuando se realizaron las encuestas, no se observó ningún patrón definido de relación con la riqueza, pero las personas de piel negra o parda presentaron un riesgo significativamente mayor que las de piel blanca. Se observaron resultados coherentes en cuanto a la educación en todas las regiones; las personas con 12 o más años de estudios presentaron un menor riesgo que las de los otros grupos.

En el cuadro 4, se indica que incluso tras el ajuste por región y estatus socioeconómico, la seroprevalencia se mantuvo significativamente mayor entre las personas indígenas, pardas y negras.

## DISCUSIÓN

El presente estudio es la mayor encuesta serológica poblacional con determinación de anticuerpos contra el SARS-CoV-2 en los países de ingresos bajos y medianos, y solo es comparable con las encuestas nacionales realizadas en España (4). Nuestros hallazgos indican que la pandemia de COVID-19 está afectando con mayor intensidad a los grupos de población más pobres y desfavorecidos de Brasil. La proporción de pruebas con resultado positivo fue mayor entre las personas indígenas, negras y pardas por comparación con las personas blancas, y se observó una relación inversa con la posición socioeconómica.

**CUADRO 1.** Distribución de la muestra del estudio según las características socioeconómicas y demográficas

	Muestra del estudio		Población brasilera 2019 (%)
	Número	%	
Región Norte Noreste Sureste Sur Centro-oeste	16 013 26 809 21 860 14 888 9 792	17,9 29,9 24,5 16,7 11,0	8,8 27,2 42,1 14,3 7,8
Sexo Masculino Femenino	37 309 52 053	41,8 58,2	51,7 48,3
Edad (años) ≤9 10-19 20-39 40-59 ≥60	4 263 8 024 27 485 28 402 21 188	4,8 9,0 30,8 31,7 23,7	12,9 15,3 33,2 24,8 13,7
Color de la piel o etnia Blanco Pardo Negro Asiático Indígena	32 383 40 088 11 304 2 446 1 219	37,0 45,9 12,9 2,8 1,4	45,2 45,1 8,9 0,5 0,4
Escolaridad (años)^a^ ≤4 5-8 9-11 ≥12	14 128 13 208 28 315 17 013	19,4 18,2 39,0 23,4	
Quintil de riqueza Más pobre Segundo Tercero Cuarto Más rico	20 976 16 560 17 325 17 262 17 230	23,5 18,5 19,4 19,3 19,3	
Total	89 362^b^		

***Fuente*** preparado por los autores a partir de los resultados.

a Se excluyeron a las personas menores de 20 años.

b En el caso del color de la piel y el grupo étnico, el total no suma 89 362 debido a la falta de datos (n = 1 922); y en el caso de los quintiles de riqueza falta la información de 9 personas. Con respecto a la escolaridad, faltaron los datos de 4 411 personas.

En cuanto a las desigualdades étnicas en la salud y nutrición en el país, varios estudios han revelado una mayor mortalidad en los niños y adolescentes indígenas que en otros grupos étnicos (11), así como diferencias similares en la mortalidad de los adultos (12). De hecho, hay pruebas abrumadoras de que las poblaciones indígenas fueron dejadas atrás cuando mejoraron las condiciones de salud en Brasil en los últimos años (13). Sería sorprendente que la COVID-19 resultara diferente de otras situaciones de salud.

Se ha informado de que la COVID-19 está azotando gravemente las aldeas indígenas rurales ubicadas en las reservas (14), pero no hay comparaciones con otros grupos étnicos. Como se señaló en la introducción, Baqui et al. encontraron que en Brasil la mortalidad hospitalaria por COVID-19 era mayor en las personas con ascendencia negra o mixta que en las blancas (5). Ese estudio se basó en un conjunto de datos públicos sobre las hospitalizaciones, pero debido a que solo unas cuantas personas se identificaron como indígenas, no se estimó la mortalidad entre ellas (5).

Dado que el grado de relación entre la seroprevalencia de la COVID-19 y el color de la piel disminuyó tras el ajuste por región del país, se determinó que el lugar de residencia había inflado en parte la diferencia de cinco veces mayor entre las personas indígenas y blancas en cuanto a la seroprevalencia de anticuerpos. Sin embargo, incluso después del ajuste por región la probabilidad de que las personas indígenas presentaran anticuerpos contra el SARS-CoV-2 fue de aproximadamente el doble que en las blancas, y en los análisis nacionales con ajuste por región y estatus socioeconómico, el índice de prevalencia se mantuvo cercano a dos.

La interpretación de estos análisis indica que las personas indígenas presentaron un riesgo sustancialmente mayor que otros grupos étnicos, en parte debido a que están concentradas en la región amazónica, donde la prevalencia era la más elevada del país en el momento de la encuesta, y también porque tienen el nivel de vida más bajo en comparación con otros grupos. El mayor riesgo de las personas indígenas se mantuvo en los análisis estratificados y ajustados por estatus socioeconómico. Los futuros estudios deberán investigar los mecanismos que sustentan esta relación.

**CUADRO 2.** Prevalencia de anticuerpos contra el SARS-CoV-2 según las características socioeconómicas y demográficas

	Número de muestras positivas	Seroprevalencia (%)	Índice de prevalencia (IC95%)		
			Sin ajustar	Ajustado por sexo y edad	Ajustado por sexo, edad y región
Región Norte Noreste Sureste Sur Centro-oeste	1 065 776 149 31 43	p <0,001^b^ 6,7 2,9 0,7 0,2 0,4	31,94 (21,09-48,38) 13,90 (9,14-21,14) 3,27 (2,10-5,11) Referencia (1) 2,11 (1,28-3,48)	p <0,001^b^ 33,26 (21,94-50,41) 14,15 (9,30-21,52) 3,27 (2,10-5,10) Referencia (1) 2,14 (1,29-3,52)	
Quintil de riqueza Más pobre Segundo Tercero Cuarto Más rico	621 449 389 369 236	p <0,001^c^ 3,0 2,7 2,3 2,1 1,4	2,16 (1,86-2,52) 1,98 (1,68-2,33) 1,64 (1,40-1,92) 1,56 (1,33-1,84) Referencia (1)	p <0,001 2,16 (1,86-2,51) 1,98 (1,69-2,31) 1,64 (1,39-1,92) 1,56 (1,33-1,83) Referencia (1)	p <0,001^b^ 1,43 (1,23-1,66) 1,48 (1,27-1,73) 1,39 (1,19-1,63) 1,44 (1,23-1,69) Referencia (1)
Escolaridad (años)^a^ ≤4 5-8 9-11 ≥12	326 347 722 253	p <0,001^c^ 2,3 2,6 2,6 1,5	1,55 (1,31-1,83) 1,77 (1,49-2,09) 1,71 (1,49-1,98) Referencia (1)	p <0,001^c^ 1,84 (1,54-2,20) 1,90 (1,61-2,26) 1,71 (1,48-1,97) Referencia (1)	p <0,001^c^ 1,46 (1,24-1,75) 1,72 (1,46-2,03) 1,46 (1,27-1,68) Referencia (1)
Color de la piel o etnia Blanco Pardo Negro Asiático Indígena	372 1 237 282 52 66	p <0,001^b^ 1,2 3,1 2,5 2,1 5,4	Referencia (1) 2,69 (2,39-3,02) 2,17 (1,86-2,53) 1,85 (1,38-2,48) 4,71 (3,65-6,08)	p <0,001^b^ Referencia (1) 2,71 (2,41-3,05) 2,18 (1,87-2,54) 1,86 (1,39-2,49) 4,73 (3,67-6,11)	p <0,001^b^ Referencia (1) 1,49 (1,32-1,68) 1,36 (1,16-1,59) 1,10 (0,82-1,49) 2,25 (1,74-2,90)

***Fuente:*** preparado por los autores a partir de los resultados.

a Se excluyeron a las personas menores de 20 años.

b Prueba de heterogeneidad.

c Prueba de tendencia lineal.

En cuanto al grupo étnico, la segunda prevalencia más elevada correspondió a la categoría de moreno o pardo, que incluye a las personas que informaron tener ascendencia mixta. Los estudios de ascendencia genómica (15) indican que en la ciudad norteña de Belém las personas que se autoclasificaron como pardas tenían en promedio 69% de ascendencia europea, 21% de ascendencia amerindia y 11% de ascendencia africana, mientras que en el sur tenían en promedio 44%, 11% y 45% de ascendencia europea, amerindia y africana, respectivamente. Por consiguiente, la evidencia indica que en norte del país las personas pardas, que presentaron una seroprevalencia del 7,1%, están genéticamente más cerca de los amerindios que las personas pardas de otras partes del país.

Con respecto a las limitaciones del estudio, se ha criticado el uso de pruebas serológicas rápidas en la toma de decisiones clínicas y para determinar quiénes poseen inmunidad al virus de la COVID-19. Sin embargo, la utilización de este tipo de pruebas para estimar la seroprevalencia es mucho menos controvertida, siempre y cuando la prueba haya sido validada (16, 17). La prueba rápida utilizada en el presente estudio (prueba de anticuerpos contra el SARS-CoV-2 de Wondfo) se sometió a diferentes estudios de validación que emplearon la RT-PCR como técnica de referencia, incluido un estudio realizado por nuestro equipo. Estos estudios estimaron que la sensibilidad y especificidad de la prueba es de 84,8% y 99,0%, respectivamente. Se ha indicado que el uso de sangre capilar para estimar la seroprevalencia tiende a aumentar la tasa de resultados negativos falsos (18), pero esto no se ha reproducido en otros estudios (19). Cabe señalar que en nuestro estudio de validación (8) se utilizó sangre capilar y la sensibilidad observada fue similar a la notificada en otros estudios. Por lo tanto, esto no debe considerarse como una limitación importante del estudio, ya que todos los subgrupos de población debieran verse afectados. Los últimos datos científicos indican que la concentración de anticuerpos contra el SARS-CoV-2 disminuye rápidamente al cabo de unas cuantas semanas, independientemente del tipo de prueba utilizada (20); por consiguiente, nuestros resultados corresponden a infecciones relativamente recientes, más que a la prevalencia acumulada; y, nuevamente, es probable que todos los subgrupos de población se vean afectados de manera similar.

La restricción de la muestra a sitios centinela que son las ciudades más grandes y desarrolladas no debe considerarse una limitación importante, ya que la intención no es estimar la prevalencia de la infección en todo el país, sino su relación con las características socioeconómicas y demográficas.

Debido a dificultades logísticas, incluida una campaña masiva de noticias falsas en las redes sociales, no fue posible completar la primera ronda de la encuesta en 87 ciudades, por lo que el tamaño de la muestra fue de 25 025 en lugar de las 33 250 pruebas previstas. Estas dificultades se superaron en las dos siguientes rondas, donde casi se alcanzó el tamaño previsto de la muestra.

En cuanto al sesgo de selección, las tasas de respuesta de alrededor del 54% son similares a las registradas en la encuesta española (59,5%) y superiores a las alcanzadas en las encuestas nacionales realizadas en Islandia y Austria, cuyas tasas de respuesta fueron aproximadamente una tercera parte de la muestra prevista (21). La mayor proporción de mujeres en la muestra estudiada podría deberse al hecho de que los hombres fueron menos propensos a cumplir la recomendación de quedarse en casa. La razón más frecuente de la falta de respuesta fue que toda la familia estuviera fuera de casa cuando tenía lugar la visita, lo que quizá se debió a que se trasladaban temporalmente a ciudades más pequeñas o zonas rurales, ya que en la primera etapa de la pandemia las ciudades de mayor tamaño se vieron más gravemente afectadas. Con respecto a las poblaciones indígenas, cabe señalar que la muestra del presente estudio se limitó a los residentes de las zonas urbanas. Por último, la muestra tuvo menos niños de lo esperado, probablemente debido a su renuencia a recibir un pinchazo en el dedo cuando eran seleccionados al azar; en estos casos, una segunda persona era seleccionada al azar y si esa persona también se negaba, se pasaba a otro hogar.

**CUADRO 3.** Prevalencia e índice de prevalencia de anticuerpos contra el SARS-CoV-2 según las características socioeconómicas y demográficas, estratificadas por región

	Resultados por región del país											
	Norte				Noreste				Sureste, Sur y Centro-oeste			
	N	Muestras positivas		Índice de prevalencia sin ajustar (IC95%)	N	Muestras positivas		Índice de prevalencia sin ajustar (IC95%)	N	Muestras positivas		Índice de prevalencia sin ajustar (IC95%)
		N	%			%	N			N	%
Quintil de riqueza Más pobre Segundo Tercero Cuarto Más rico	4 985 3 243 2 820 2 577 2 379	355 218 184 184 124	p = 0,03^b^ 7,1 6,7 6,5 7,1 5,2	1,37 (1,11-1,68) 1,29 (1,03-1,61) 1,25 (1,02-1,54) 1,37 (1,09-1,72) Referencia (1)	7 788 5 772 5 262 4 404 3 583	231 194 156 128 67	p = 0,004^b^ 3,0 3,4 3,0 2,9 1,9	1,59 (1,21-2,08) 1,80 (1,35-2,39) 1,59 (1,19-2,12) 1,55 (1,16-2,08) Referencia (1)	8 203 7 545 9 243 10 281 11 268	35 37 49 57 45	p = 0,38^c^ 0,4 0,5 0,5 0,6 0,4	1,07 (0,68-1,68) 1,23 (0,80-1,88) 1,33 (0,88-2,01) 1,39 (0,96-2,01) Referencia (1)
Escolaridad (años)^a^ ≤4 5-8 9-11 ≥12	2 167 2 020 5 393 2 682	159 157 375 149	p = 0,01^b^ 7,3 7,8 7,0 5,6	1,32 (1,05-1,66) 1,40 (1,12-1,75) 1,25 (1,04-1,50) Referencia (1)	4 581 3 859 8 782 3 969	139 149 256 71	P <0,001^b^ 3,0 3,9 2,9 1,8	1,70 (1,29-2,23) 2,16 (1,63-2,95) 1,63 (1,27-2,10) Referencia (1)	7 380 7 329 14 140 10 362	28 41 91 33	p = 0,002^c^ 0,4 0,6 0,6 0,3	1,19 (0,70-2,04) 1,76 (1,08-2,85) 2,02 (1,34-3,04) Referencia (1)
Color de piel y etnia Blanco Pardo Negro Asiático Indígena	2 729 10 052 1 954 524 400	133 730 116 20 42	p <0,001^c^ 4,9 7,3 5,9 3,8 10,5	Referencia (1) 1,49 (1,26-1,76) 1,22 (0,98-1,51) 0,78 (0,50-1,22) 2,15 (1,58-2,93)	6 418 14 048 4 400 838 393	158 410 131 27 22	p = 0,01^c^ 2,5 2,9 3,0 3,2 5,6	Referencia (1) 1,19 (0,98-1,44) 1,21 (0,95-1,54) 1,31 (0,86-2,00) 2,27 (1,44-3,60)	23 236 15 988 4 950 1 084 426	81 97 35 5 2	p = 0,002^c^ 0,4 0,6 0,7 0,5 0,5	Referencia (1) 1,74 (1,28;2,38) 2,03 (1,33;3,09) 1,32 (0,54-3,24) 1,35 (0,33-5,47)

***Fuente*:** Preparado por los autores a partir de los resultados.

a Se excluyeron a las personas menores de 20 años.

b Prueba de tendencia lineal.

c Prueba de heterogeneidad.

**CUADRO 4.** Índice de prevalencia de anticuerpos contra el SARS-CoV-2 según el color de la piel y la etnia

	Índice de prevalencia (IC95%)		
Color de piel o etnia	Sin ajustar	Ajustado por región	Ajustado por región y quintil de riqueza
Blanco Pardo Negro Asiático Indígena	p <0,001^a^ Reference (1) 2,69 (2,39-3,02) 2,17 (1,86-2,53) 1,85 (1,38-2,48) 4,71 (3,65-6,08)	p <0,001^a^ Reference (1) 1,46 (1,30-1,65) 1,35 (1,16-1,58) 1,10 (0,82-1,48) 2,25 (1,74-2,91)	p <0,001^a^ Reference (1) 1,43 (1,27-1,62) 1,32 (1,13-1,54) 1,08 (0,81-1,45) 2,17 (1,68-2,81)

***Fuente*:** preparado por los autores a partir de los resultados.

a Prueba de heterogeneidad.

En resumen, con los análisis de las tres rondas de encuestas serológicas nacionales realizadas en Brasil se revelaron importantes desigualdades en la prevalencia de anticuerpos contra el SARS-CoV-2 según la riqueza familiar, la educación y el grupo étnico. Contrariamente a las primeras impresiones de que la COVID-19 afectaría a todos los grupos de la sociedad brasileña con una intensidad similar, con nuestros análisis se demuestra que las personas de familias pobres y con poca escolaridad se encontraban expuestas a un mayor riesgo de infección. En cuanto al grupo étnico y el color de la piel, las personas blancas presentaron el menor riesgo, mientras que las personas de la población indígena, negra y parda fueron las más afectadas (22).

## Declaración.

Los autores son los únicos responsables de las opiniones expresadas en el artículo, que no necesariamente reflejan el criterio o la política de la *RPSP/PAJPH* o de la OPS.
